# Darutoside ameliorates adjuvant-induced arthritis in mice by repolarizing M1/M2 macrophages through inhibition of JAK2-STAT3-p65 signaling pathway

**DOI:** 10.1515/biol-2025-1357

**Published:** 2026-08-10

**Authors:** Jun Wang, Shiya Gao, Mingsong Meng, Zilin Zhang, Sen Zhang, Jing Han, Dongsheng Wang

**Affiliations:** School of Pharmacy, Nanjing University of Chinese Medicine, Nanjing, 210023, China; Nanjing Hospital of Chinese Medicine Affiliated to Nanjing University of Chinese Medicine, Nanjing, 210022, China; Department of Spine Surgery, Center of Orthopedics, Daping Hospital, Army Medical University (Third Military Medical University), Chongqing Key Laboratory of Spinal Disease Therapy and Regeneration (Military-Civilian), Chongqing, 400042, China

**Keywords:** rheumatoid arthritis, macrophages, darutoside, JAK2-STAT3

## Abstract

Darutoside (DA), a diterpenoid compound derived from *Herba Siegesbeckiae*, possesses notable anti-inflammatory properties. This study evaluated the effectiveness of DA in treating rheumatoid arthritis (RA) symptoms and elucidated the relevant mechanisms. The anti-RA efficacy of DA was investigated in a Complete Freund’s Adjuvant (CFA)-induced arthritis mouse model, while RAW264.7 macrophages were used to examine DA’s impact on macrophage polarization *in vitro*. Targets of DA for RA were predicted through protein-protein interaction networks and pathway enrichment analysis. Subsequently, mechanisms were verified through *in vitro* assays. The results showed that DA alleviated the symptoms in the CFA mouse model, as evidenced by reduced clinical scores, attenuated paw swelling, and decreased inflammatory response. Moreover, DA induced repolarization of M1/M2 macrophages in both the CFA model mice and RAW264.7 cells. Network pharmacology analysis suggested that DA may exert its anti-RA effects by modulating the JAK-STAT signaling pathway. Western blotting confirmed that DA inhibited the JAK2-STAT3-p65 pathway, thereby suppressing M1 macrophage polarization. Furthermore, the repolarization effect of DA on macrophages was counteracted in JAK2-silenced cells. These findings indicate that DA attenuates arthritis symptoms in CFA mice, and this effect may be attributed to its regulation of macrophage M1/M2 polarization through suppression of the JAK2-STAT3 pathway and downstream p65 activation.

## 1 Introduction

Rheumatoid arthritis (RA) is an autoimmune disorder characterized by chronic joint pain, swelling, deformity, and functional impairment resulting from dysregulated immune responses [1]. The intricate interplay between genetic and environmental factors contributes to immune dysfunction, synovitis, and joint destruction [2]. Current treatments for RA include pharmacotherapy, physical therapy, rehabilitation, and dietary intervention. Commonly used pharmaceutical agents comprise glucocorticoids (GCs), disease-modifying antirheumatic drugs (DMARDs), and nonsteroidal anti-inflammatory drugs (NSAIDs) [3]. While these drugs alleviate symptoms and improve disease control, they often fail to halt disease progression and may cause significant adverse effects, thereby limiting their clinical utility [4]. Consequently, novel and effective anti-RA drugs are urgently needed.

*Herba Siegesbeckiae*, also known as “Xi-Xian Cao,” is a commonly employed herb in traditional Chinese medicine for its anti-rheumatic properties [5]. Previous studies have identified luteolin as an active compound in *Herba Siegesbeckiae* targeting the TLR4 pathway in RA [6]. Darutoside (DA), another bioactive diterpenoid from the same herb [7], has been shown to relieve symptoms in acute gouty arthritis and inhibit ear edema in animal models, indicating potential anti-inflammatory effects relevant to diseases like RA [7], 8]. However, unlike luteolin, the therapeutic potential of DA in RA remains unclear.

Macrophages exhibit inherent plasticity, allowing them to adapt to diverse environmental cues and perform various functions [9]. They can be polarized into classical (M1) and alternative (M2) activation subsets. M1 macrophages release pro-inflammatory cytokines such as TNF-α and IL-1β, whereas M2 macrophages release anti-inflammatory mediators such as IL-10 and arginase-1 (Arg-1) [10]. In the context of RA, macrophage polarization shifts towards the M1 phenotype, with concomitant inhibition of M2 polarization, leading to inflammation by promoting pro-inflammatory molecules and suppressing anti-inflammatory factors, thereby contributing to synovitis and bone erosion and exacerbating patient symptoms [11]. Therefore, manipulating macrophage polarization by suppressing M1 and promoting M2 polarization holds promise as a strategy for RA treatment [12].

In the present study, we extensively investigated the anti-RA potential of DA using a Complete Freund’s Adjuvant (CFA)-induced arthritis mouse model. Additionally, we investigated its effects on macrophage polarization (M1/M2) and the underlying molecular mechanisms. These findings may contribute to the development of novel therapeutic strategies for RA.

## 2 Materials and methods

### 2.1 Reagents

DA was purchased from Yuanyebio (purity > 98 %, China). Isoflurane was obtained from RWD Life Science (China), and Complete Freund’s Adjuvant (CFA) was sourced from Chondrex (USA). Fetal bovine serum (FBS) and Dulbecco’s Modified Eagle Medium (DMEM) were obtained from Gibco (USA). Lipopolysaccharide (LPS) was purchased from Sigma (USA). Interferon-γ (IFN-γ), interleukin-13 (IL-13), and interleukin-4 (IL-4) were obtained from Proteintech (USA). CCK-8 kit was sourced from APEXBIO (USA). TRIzol reagent and SYBR RT-qPCR kit were purchased from Takara (Japan), while BCA protein assay kits were from Beyotime (China). Antibodies including Alexa647-conjugated CD206, PE-Cy7-conjugated CD86, BV421-conjugated F4/80, and purified rat anti-mouse CD16/CD32 (mouse BD Fc block™) were from BD Pharmingen (USA). Fluor647-conjugated goat anti-mouse IgG (H + L) was sourced from Affinity (China), and FITC-conjugated AffiniPure goat anti-rabbit IgG (H + L) was obtained from Proteintech (USA). Antibodies for JAK2, p-JAK2, STAT3, p-STAT3 (Phospho-Y705), p-p65, p65, and HRP-conjugated goat anti-rabbit IgG were all sourced from Cell Signaling Technology (USA). JAK2 siRNA and Lipofectamine 2000, along with all primers, were supplied by Sangon Biotech (China).

### 2.2 Animals

Male C57BL/6 mice (weighing 18–22 g and aged 6–8 weeks) were obtained from Vital River Laboratory Animal Technology Co., Ltd. They were maintained in a controlled environment with a temperature of 22–26 °C, a humidity of 55–60 %, a 12-h light/dark cycle, unrestricted access to purified water, and a standard pellet diet (Jiangsu Xietong Biomedical Engineering, China).

**Ethical approval:** The research related to animal use complied with all the relevant national regulations and institutional policies for the care and use of animals, and has been approved by the Animal Experiment Ethics Committee of the Nanjing University of Chinese Medicine (Approval number: 202203A084).

### 2.3 CFA-induced murine arthritis model

Mice were anesthetized with isoflurane and then injected with 20 μL CFA (4 mg/mL) into the plantar surface of their left hind paw. Controls received an equivalent volume of saline [13]. Dexamethasone (DM), a classic drug for RA treatment, was used as the positive control drug [14]. Previous research has shown that DA can suppress symptoms at 0.2–2 mg/kg/day in a xylene-induced-ear edema mouse model [7]. Following the administration of CFA, the mice were administered either vehicle, DA (0.2 or 2 mg/kg), or DM (2 mg/kg) orally daily for 27 days. Paw and ankle thickness, arthritis severity, and body weight were evaluated in a blinded manner throughout the trial. The thickness of the left hind paw and ankle joint was measured using an electronic caliper, and arthritis severity was graded on a scale of 0–4 [15].

### 2.4 Cell culture, transfection, and grouping

RAW264.7 cells obtained from the Chinese National Center for Cell Culture Identification were cultured in DMEM supplemented with 1 % penicillin-streptomycin and 10 % fetal bovine serum at 37 °C under 5 % CO_2_. For dose-response analysis, when cells reached 70–80 % confluence, they were pretreated with various concentrations of DA (0.3, 0.6, and 1.2 μM) or vehicle control for 2 h, followed by stimulation with LPS (100 ng/mL) plus IFN-γ (20 ng/mL) for 30 min before protein harvesting. Total protein was extracted and subjected to Western blot analysis for phosphorylated and total forms of JAK2, STAT3, and p65. For siRNA transfection experiments, when cell confluence reached 50–60 %, JAK2-specific siRNA or control siRNA (Table 1) was transfected using Lipofectamine 2000 according to the manufacturer’s recommended protocol. Following a 6-h transfection period, the medium was replaced with antibiotic-free DMEM, and cells were incubated for an additional 24 h. Subsequently, cells were pretreated with DA or vehicle for 2 h, followed by stimulation with LPS (100 ng/mL) plus IFN-γ (20 ng/mL) for 30 min, after which protein was harvested for Western blot analysis of phosphorylated signaling molecules. The experimental groups for siRNA experiments were as follows: (1) control siRNA; (2) JAK2-siRNA; (3) LPS + IFN-γ + control siRNA; (4) LPS + IFN-γ + JAK2-siRNA; (5) DA + LPS + IFN-γ + control siRNA; and (6) DA + LPS + IFN-γ + JAK2-siRNA.

**Table 1:** siRNA oligonucleotide sequence.

SiRNA	Oligonucleotide sequence	
Negative control	Sense	5ʹ-UUC​UCC​GAA​CGU​GUC​ACG​UTT-3ʹ
	Antisense	5ʹ-ACG​UGA​CAC​GUU​CGG​AGA​ATT-3ʹ
Mouse JAK2 siRNA	Sense	5ʹ-GAG​CUU​UGG​AGU​GGU​UCU​AUA​TT-3ʹ
	Antisense	5ʹ-UAU​AGA​ACC​ACU​CCA​AAG​CUC​TT-3ʹ

### 2.5 Cell viability

RAW264.7 cells (2 × 10^4^ cells/well) were plated in 96-well plates and cultured for 24 h. Subsequently, different doses of DA were applied to respective wells with specific incubation durations (24 or 48 h). After this period, each well received 10 μL of CCK-8, followed by incubation for 2 h, after which absorbance was measured at 450 nm using a microplate reader.

### 2.6 Quantitative real-time PCR (qRT-PCR)

Cultured cells or synovial tissues from mouse ankle joints were processed with TRIzol reagent (Takara) for RNA isolation. The extracted RNA was reverse transcribed into cDNA using PrimeScript RT Master Mix (Q711-02, Vazyme) and subsequently analyzed *via* qRT-PCR to quantify the mRNA levels of the target genes. Results were calculated using the 2^−ΔΔCt^ method [15], with the primer sequences provided in Table 2.

**Table 2:** Primers used for qRT-PCR analysis.

Oligo sets mouse	Pimer (5′ to 3′)
*Gapdh*	Forward: AGG​TCG​GTG​TGA​ACG​GAT​TTG
	Reverse: TAG​ACC​ATG​TAG​TTG​AGG​TCA
*Tnf-α*	Forward: GAC​CCT​CAC​ACT​CAG​ATC​AT
	Reverse: TTG​AAG​AGA​ACC​TGG​GAG​TA
*Il-1β*	Forward: CAA​CCA​ACA​AGT​GAT​ATT​CTC​CAT​G
	Reverse: GAT​CCA​CAC​TCT​CCA​GCT​GCA
*Il-6*	Forward: GAG​GAT​ACC​ACT​CCC​AAC​AGA​CC
	Reverse: AAG​TGC​ATC​ATC​GTT​GTT​CAT​ACA
*Inos*	Forward: AAGCCCTCACCTACTTCC
	Reverse: CTGAGGGCTGACACAAGG
*Cd86*	Forward: TTC​GGC​TTC​TTG​TGA​CAT​AC
	Reverse: ACG​GAG​TCA​ATG​AAG​ATT​TCC​T
*Il-10*	Forward: AAG​GCA​GTG​GAG​CAG​GTG​AA
	Reverse: CCA​GCA​GAC​TCA​ATA​CAC​AC
*Cd206*	Forward: CTC​TGT​TCA​GCT​ATT​GGA​CGC
	Reverse: CGG​AAT​TTC​TGG​GAT​TCA​GCT​TC
*Arg1*	Forward: CTC​CAA​GCC​AAA​GTC​CTT​AGA​G
	Reverse: AGG​AGC​TGT​CAT​TAG​GGA​CAT​C

### 2.7 ELISA analysis

At the end of the experiment, blood collected from the retro-orbital plexus was centrifuged at 3,000 rpm and 4 °C for serum isolation. The levels of cytokines, including IL-10, IL-1β, IL-6, and TNF-α, were evaluated using the specified ELISA kit from Elabscience (China), following the manufacturer’s instructions.

### 2.8 Immunofluorescence staining (IF)

IF staining was performed on RAW264.7 cells adherent to 12-mm glass coverslips. The cells were exposed to specific reagents for 24 h at 37 °C, fixed in 4 % PFA, permeabilized with 0.1 % Triton X-100, and blocked with BSA. Cells were subsequently incubated with primary antibodies against CD86 or CD206 for 12 h at 4 °C, washed with PBS, and exposed to secondary antibodies for 2 h. Cells were counterstained with DAPI for 5 min before visualization under confocal microscopy.

### 2.9 Flow cytometry (FCM)

Flow cytometry was performed as previously reported [16]. Following incubation, the cells were suspended in PBS, labeled with anti-F4/80 (ab186073, Abcam), anti-CD206 (60143-1-Ig, Proteintech), or anti-CD86 (DF6332, Affinity) antibodies, and kept in the dark on ice for 30 min. Cells were washed in PBS before examination using an acoustic focusing cytometer and FlowJo software.

### 2.10 Network pharmacology

Target prediction for DA and screening of RA-related targets were performed as previously reported [17]. The canonical SMILES of DA was generated using ChemDraw and subsequently entered into Swiss Target Prediction via PubChem for target gene prediction. GeneCards, OMIM, and DisGeNET databases were employed to analyze RA-related disease targets. Venny2.1 (https://bioinfogp.cnb.csic.es/) was used to map DA and RA targets, and the intersections were imported into the STRING database as “*Homo sapiens*,” retaining only interactions with a confidence score≥ 0.4. Peripheral targets were eliminated to generate the core targets for drug therapy. These were visualized in Cytoscape 3.9.1 software using “Generate Style from Statistics.” The network’s layout was defined based on node size (Degree) and color scheme according to Degree value.

### 2.11 Western blot

Western blotting was performed as previously reported [15]. To separate the nuclear and cytoplasmic proteins of RAW264.7 cells, a nuclear and cytoplasmic protein extraction kit (Beyotime) was used to obtain the cell lysates. Cell lysate proteins were resolved by SDS-PAGE, followed by the transfer of 50 μg protein onto PVDF membranes. Membranes were then incubated with antibodies against p-p65, p65, p-JAK2, JAK2, p-STAT3, STAT3, and GAPDH at 4 °C for 12 h. After washing, the secondary antibody was applied for 2 h. Subsequently, protein bands were detected using an ECL detection system, and band intensities were quantified using ImageJ.

### 2.12 Histological staining

At the end of the experiment, mouse ankle joints were harvested, fixed in 4 % paraformaldehyde for 48 h, and decalcified in 10 % EDTA. The decalcified tissues were dehydrated through a graded ethanol series, cleared in xylene, and embedded in paraffin. Sagittal sections (5 μm thick) were mounted on poly-l-lysine-coated slides. For histopathological evaluation, sections were stained with hematoxylin and eosin (H&E) to assess synovial inflammation and tissue architecture, or with Safranin O-fast green to evaluate cartilage proteoglycan content and structural integrity, as previously reported [18]. Stained sections were examined under a light microscope, and images were captured.

### 2.13 Statistical analysis

Statistical data are presented as mean ± SD Statistical analyses were performed using GraphPad Prism 8.01 for graph generation and statistical testing. Comparisons between two groups were performed using Student’s t-test, while comparisons among multiple groups were performed using one-way ANOVA. Significance was set at *P* < 0.05.

## 3 Results

### 3.1 DA effectively alleviates CFA-induced arthritis symptoms in mice by repolarizing macrophages from M1 to M2

**Figure 1:**
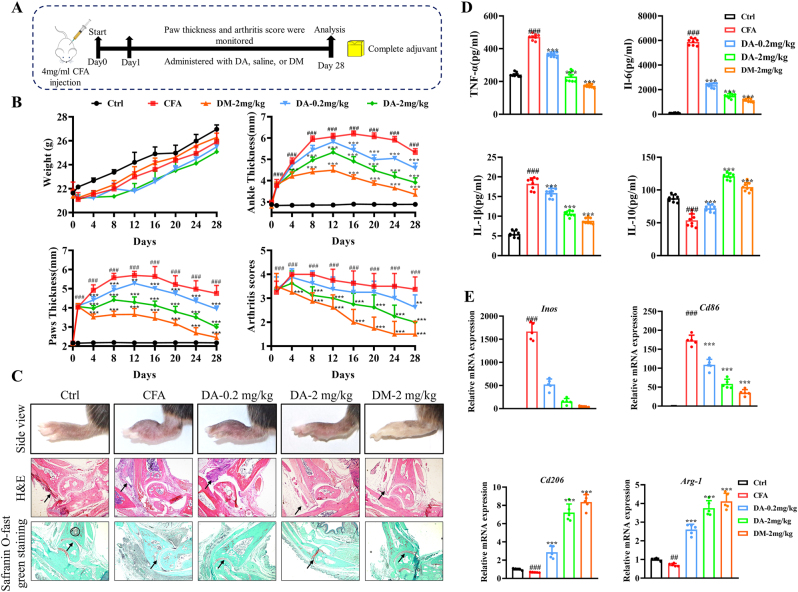
Therapeutic effects of DA on CFA mice. (A) Experimental schedule, (B) measurements of body weight, ankle thickness, paw thickness, and arthritis score of the mice, *n* = 8 per group, (C) side view, H&E staining, and Safranin O-fast green staining of hind paws illustrating the impact of DA treatment, (D) IL-1β, IL-6, TNF-α, and IL-10 levels in serum, *n* = 8 per group and (E) mRNA levels of *Inos*, *Cd86*, *Cd206*, and *Arg-1* in the paw tissue. Data represent mean ± SD from three independent experiments (*n* = 3). Statistical significance indicated as ^###^ *P* < 0.001 versus ctrl group, *P < 0.05, **P < 0.01, ***P < 0.001 versus CFA group.

To assess the therapeutic effect of DA on RA, we established a CFA-induced mouse model to mimic RA symptoms, as outlined in Figure 1A. The experiments revealed that orally administration of DA at 0.2 or 2 mg/kg significantly reduced paw thickness, ankle joint thickness, and overall arthritis score in model mice (Figure 1B). Photographs of hind paws showed marked swelling in CFA mice, which was alleviated by DA treatment (Figure 1C). Histological analysis of ankle joint sections revealed inflammatory cell infiltration and synovial hyperplasia in H&E-stained sections, as well as cartilage damage and proteoglycan loss in Safranin O-fast green-stained sections from CFA mice. Notably, DA treatment significantly ameliorated these histopathological changes (Figure 1C). Furthermore, DA decreased serum levels of pro-inflammatory cytokines (TNF-α, IL-1β, and IL-6) and increased the anti-inflammatory cytokine IL-10 (Figure 1D) in the CFA mice. In addition, DA treatment did not affect body weight compared with the CFA group (Figure 1B), indicating that DA exerted therapeutic effects without significant side effects.

The role of M1 and M2 macrophages in controlling inflammation is critical, with shifts in their pro-inflammatory cytokine production [19]. The fluctuations in serum M1- and M2-related cytokines suggest that DA may alleviate murine arthritis through macrophage repolarization. qRT-PCR analysis of ankle joint tissue mRNA levels of M1/M2 macrophage markers revealed elevated M1 markers (*Cd8*6 and *Inos*) and reduced M2 markers (*Cd206* and *Arg-1*) in the CFA group. In contrast, the DA group showed the opposite pattern (Figure 1E), suggesting that DA significantly inhibited M1 markers and slightly promoted M2 markers in the CFA model.

### 3.2 DA repolarized macrophages from M1 to M2 *in vitro*

**Figure 2:**
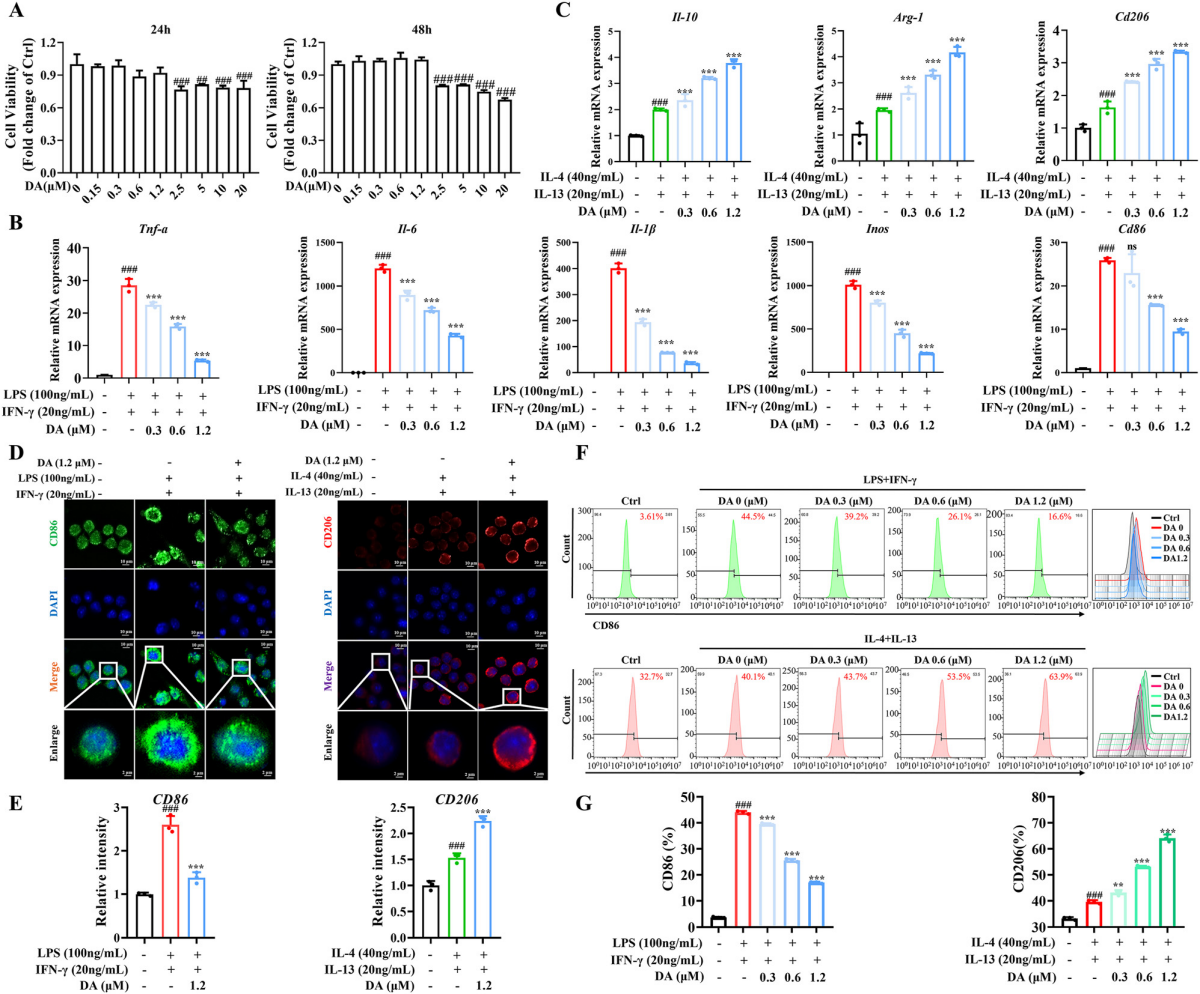
DA modulates macrophage polarization in RAW264.7 cells. (A) Cell viability of DA on RAW264.7 cells, (B) Tnf-α, Il-6, Il-1β*, Inos*, and *Cd86* mRNA levels in RAW264.7 cells, (C) Il-10, Arg-1, and *Cd206* mRNA levels in RAW264.7 cells, (D–E) immunofluorescence staining of CD86 and CD206 in RAW264.7 cells and (F–G) flow cytometry analysis of CD86^+^F4/80^+^ and CD206^+^F4/80^+^ cells. Data represent mean ± SD from three independent experiments (*n* = 3). Statistical significance designated as ^*###*^ *P* < 0.001 versus ctrl group, ***P < 0.001 versus LPS + IFN-γ or IL-4+IL-13 groups.

Using the CCK-8 assay, we evaluated the impact of DA on RAW264.7 cell viability and found negligible cytotoxicity below 1.2 μM (Figure 2A). Subsequently, we proposed utilizing 0.3, 0.6, and 1.2 μM concentrations for future experiments. Next, the influence of DA on macrophage polarization was analyzed *in vitro*. qRT-PCR analysis revealed that DA significantly attenuated LPS/IFN-γ-stimulated upregulation of M1-associated cytokines (*Tnf-α, Il-6, Il-1,β Inos*, and *Cd86*) in RAW264.7 cells (Figure 2B). Concurrently, DA slightly enhanced M2-associated markers (Il-10, Arg-1, and *Cd206*) in response to IL-4/IL-13 stimulation (Figure 2C). Immunofluorescence staining indicated that DA significantly suppressed M1-specific CD86 expression and slightly promoted M2-specific CD206 expression in RAW264.7 cells (Figure 2D and E). Flow cytometry data confirmed the capacity of DA to suppress M1 polarization and promote M2 polarization (Figure 2F and G). Overall, the findings suggest that DA may play a role in modulating macrophage polarization towards an anti-inflammatory state, particularly by inhibiting M1 macrophage polarization, thereby attenuating symptoms of RA.

### 3.3 DA suppressed JAK2-STAT3 signaling pathway to impede M1 macrophage polarization

**Figure 3:**
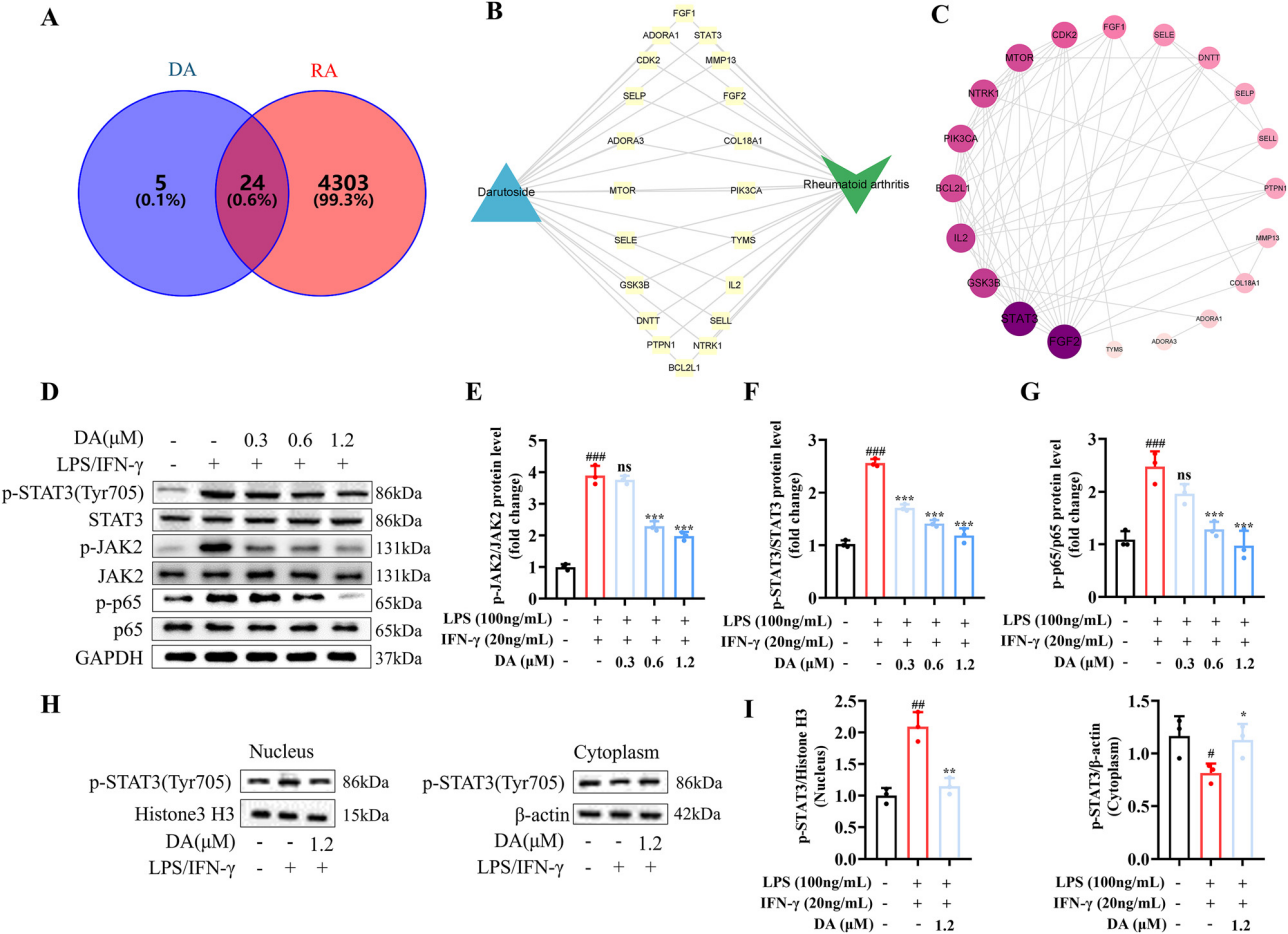
DA inhibited the protein levels of the JAK2-STAT3 pathway. (A–C) Network pharmacology prediction of DA potential targets for the treatment of RA, (D–G) p-JAK2, JAK2, p-STAT3 (Tyr705), STAT3, p-p65, and p65 protein levels in RAW264.7 cells and (H–I) p-STAT3(Tyr705), STAT3, histone H3, and β-actin proteins in the cytoplasm and nuclei of RAW264.7 cells. Data represent mean ± SD from three independent experiments (*n* = 3). Statistical significance indicated as ^*###*^ *P* < 0.001 versus ctrl group, ***P < 0.001 versus LPS + IFN-γ group.

A network pharmacology approach was conducted to explore how DA regulates macrophage polarization. We obtained the canonical SMILES of DA using ChemDraw software and retrieved 29 DA target genes from PubChem and Swiss Target Prediction. Additionally, we identified 4,327 RA-related targets through comprehensive searches in GeneCards, OMIM, and DisGeNET databases. We utilized Venny 2.1 and identified 24 intersection genes as common targets (Figure 3A). Next, we employed Cytoscape 3.8.2 to construct a “Drug-Disease-Target” network (Figure 3B). To further explore the protein-protein interactions (PPIs) relevant to the therapeutic potential of DA for RA, overlapping genes were uploaded to the STRING database, and a PPI network was constructed. FGF2, STAT3, GSK3β, IL-2, and BCL2L1 stood out among the identified core targets (Figure 3C). As STAT3 signaling is involved in macrophage polarization [20], we investigated whether DA targets the JAK-STAT3 pathway to repolarize M1 macrophages. The results showed that DA effectively attenuated the LPS/IFN-γ-induced phosphorylation of JAK2 and STAT3 in RAW264.7 cells (Figure 3D–F). In addition, compared with the control, LPS/IFN-γ significantly increased nuclear p-STAT3 (Tyr705) and decreased cytoplasmic p-STAT3 (Tyr705), while DA treatment significantly reversed these changes. Moreover, DA suppressed NF-κB pathway activation, a downstream cascade of JAK2-STAT3 [21], as evident from reduced p-p65 (Figure 3D–G).

### 3.4 DA cannot further inhibit M1 macrophage polarization in JAK2-silenced cells

**Figure 4:**
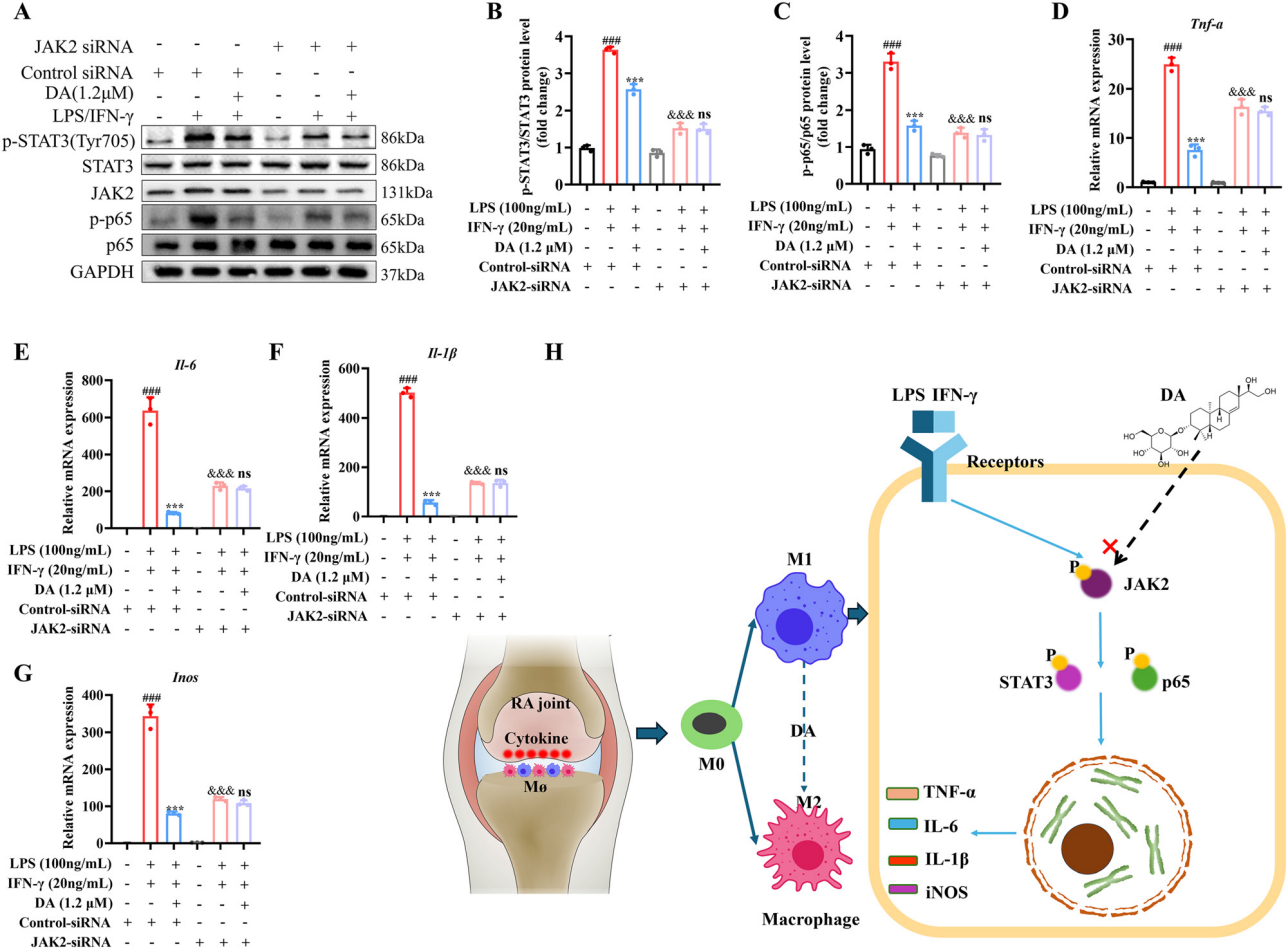
Silencing of JAK2 counteracts DA-suppressed M1 macrophage polarization. (A–C) p-STAT3, STAT3, JAK2, p-p65, and p65 protein levels in RAW264.7 cells, (D–G) Tnf-α, Il-6, Il-1β*, Inos* mRNA levels in RAW264.7 cells and (H) Schematic representation of DA’s regulation on M1 macrophage polarization *via* the JAK2-STAT3 pathway, resulting in the attenuation of symptoms in an experimental RA mouse model. Data represent mean ± SD from three independent experiments (*n* = 3). Statistical significance designated as ^*###*^ *P* < 0.001 versus ctrl group, ***P < 0.001 versus LPS + IFN-γ group, ^*&&&*^ *P* < 0.001 versus Ctrl + JAK2-siRNA group.

To delve deeper into the role of JAK2-STAT3 signaling in DA’s modulation of pro-inflammatory cytokines during M1 macrophage polarization, we designed a JAK2-targeted siRNA. After transfecting RAW264.7 cells with either a control siRNA (Ctrl) or JAK2 siRNA, we observed a profound reduction in JAK2 expression in the knockdown group, as evidenced by Western blot analysis (Figure 4A). Additionally, Western blot analysis revealed that knockdown of JAK2 eliminated the inhibitory effect of DA on p-STAT3 (Figure 4A and B) and p-p65 (Figure 4A–C). Subsequently, we quantified mRNA levels of M1 macrophage polarization markers using qRT-PCR. Notably, DA could no longer inhibit M1 markers such as *Tnf-α, Il-6, Il-1β*, and Inos (Figure 4D–G) in LPS/IFN-γ-activated JAK2-silenced RAW264.7 cells. This suggests that DA modulates M1 macrophage polarization *via* the JAK2-STAT3 signaling pathway.

## 4 Discussion

This research reveals DA’s potential therapeutic application in treating CFA-induced arthritis in mice. Mechanistically, DA prevents the upregulation of pro-inflammatory M1 cytokines and stimulates anti-inflammatory M2-related cytokines, thereby rebalancing M1/M2 macrophage polarization. Furthermore, our results suggest that DA inhibits JAK2-STAT3 signaling in RAW264.7 cells, thereby regulating macrophage function. Notably, silencing JAK2 diminished the inhibitory effect of DA on STAT3 activity, negating DA’s capacity to suppress M1-related factors. These data confirm that DA inhibits the JAK2-STAT3 signaling pathway to hinder M1 macrophage polarization, thereby ultimately mitigating murine arthritis.

*Herba Siegesbeckiae* has demonstrated anti-allergic, anti-inflammatory, antioxidant, antibacterial, and antithrombotic effects. Historically, it has been used for rheumatic pain, lumbosacral weakness, knee discomfort, and limb numbness [5]. Phytochemical studies have identified three principal compound categories: diterpenoids, sesquiterpenoids, and flavonoids, with diterpenoids as the primary component [22]. Within these diterpenoids, DA emerged as an anti-inflammatory and analgesic constituent of *Herba Siegesbeckiae* [7]. Our study found that DA notably improved CFA-induced arthritis symptoms in mice, reducing paw swelling and arthritis scores. Notably, a high dose of DA achieved a therapeutic effect comparable to DM in CFA mice. Moreover, DA displayed negligible toxicity based on animal weight monitoring. These results suggest that DA may serve as a safe and effective therapy for RA.

Emerging research highlights innate immunity’s crucial role in regulating RA pathogenesis [23]. Macrophages play a vital role in this system, and their initial increase in the synovium indicates RA onset [24]. Macrophages can adapt to different microenvironments and can be categorized into M1 or M2 subtypes. M1 macrophages produce inflammatory cytokines (e.g., IL-1β, TNF-α), while M2 macrophages release anti-inflammatory mediators like IL-10 [24]. After the onset of RA, various factors contribute to an increase in the number of M1 macrophages, whose production of IL-1β and TNF-α contributes to synovitis and bone erosion. Therefore, shifting macrophage polarization from M1 to M2 dominance presents a potential approach for managing RA [25]. Our research demonstrated that DA treatment significantly reduced the pro-inflammatory cytokines associated with M1 macrophages in both ankle tissue and serum of CFA-induced arthritis mice *in vivo* and LPS/IFN-γ-activated RAW264.7 cells *in vitro*. Additionally, DA slightly increased levels of anti-inflammatory cytokines tied to M2 macrophages in CFA mice and IL-4/IL-13-stimulated RAW264.7 cells. This suggests that DA’s anti-RA action primarily involves inhibiting M1 macrophage polarization while slightly promoting M2 macrophage polarization.

Macrophage polarization involves complex signaling networks, including JAK-STAT, PI3K-AKT, JNK, and Notch [26], 27]. Following cytokine-receptor engagement that activates JAK tyrosine kinases (JAK1, JAK2, JAK3, and TYK2), receptor phosphorylation, STAT activation, and downstream gene expression profoundly influence macrophage polarization [28]. Therefore, inhibiting JAK-STAT signaling to suppress inflammatory factor secretion and maintain macrophage homeostasis is a critical therapeutic strategy for RA [28]. As STAT3 is known to directly bind to the transactivation domain of NF-κB p65 [21], our study demonstrated that DA inhibits the JAK2-STAT3 pathway, as evidenced by reduced p-JAK2, p-STAT3, and p-p65 levels in LPS/IFN-γ-stimulated macrophages.

To further elucidate whether DA’s effects on macrophage polarization are predominantly mediated through JAK2, we employed siRNA to silence JAK2 expression. Our findings revealed that DA failed to further inhibit M1 polarization or exert additional anti-inflammatory effects in JAK2-silenced macrophages, suggesting that JAK2 is a principal, if not indispensable, mediator of DA’s action. Moreover, the concomitant reduction of p-p65 upon JAK2 silencing indicated that p65 phosphorylation occurs downstream of JAK2-STAT3 activation, potentially through STAT3-mediated transcriptional regulation of NF-κB components or direct STAT3-p65 physical interaction [21]. Notably, the therapeutic effect of DA alone was slightly superior to that of the JAK2 knockdown (si-JAK2) group, suggesting that DA likely targets multiple pathways or possesses additional mechanisms beyond JAK2-STAT3-p65, contributing to its overall therapeutic efficacy through cooperative interaction with JAK2-STAT3 signaling or via JAK2-independent mechanisms. Collectively, these results suggest that DA predominantly inhibits M1 macrophage polarization by suppressing JAK2-STAT3 signaling pathway-mediated transcriptional activation, thereby reducing inflammation and alleviating RA symptoms.

## 5 Conclusions

This work provides experimental evidence of DA’s efficacy in RA treatment. Specifically, DA’s anti-RA effect is attributed to its regulation of the M1/M2 macrophage polarization balance *via* the JAK2-STAT3 signaling pathway (Figure 4H). Further study should investigate DA’s influence on other cellular components within the synovial microenvironment, such as synovial fibroblasts, *B* cells, and *T* cells.
